# Synthesis and Pharmacological Evaluation of a Novel Peptide Based on *Anemonia sulcata* BDS-I Toxin as a New K_V_3.4 Inhibitor Exerting a Neuroprotective Effect Against Amyloid-β Peptide

**DOI:** 10.3389/fchem.2019.00479

**Published:** 2019-07-09

**Authors:** Roselia Ciccone, Ilaria Piccialli, Paolo Grieco, Francesco Merlino, Lucio Annunziato, Anna Pannaccione

**Affiliations:** ^1^Division of Pharmacology, Department of Neuroscience, Reproductive and Dentistry Sciences, School of Medicine, Federico II University of Naples, Naples, Italy; ^2^Department of Pharmacy, University of Naples Federico II, Naples, Italy; ^3^Fondazione IRCSS SDN Napoli, Naples, Italy

**Keywords:** BDS-I, voltage gated potassium channel, K_V_3.4, Alzheimer's disease, Aβ peptide

## Abstract

There is increasing evidence that the fast-inactivating potassium current I_A_, encoded by K_V_3. 4 channels, plays an important role in Alzheimer's Disease (AD), since the neurotoxic β-amyloid peptide1-42 (Aβ_1−42_) increases the I_A_ current triggering apoptotic processes. The specific inhibition of K_V_3.4 by the marine toxin extracted from *Anemonia sulcata*, named blood depressing substance-I (BDS-I), reverts the Aβ peptide-induced cell death. The aim of the present study was to identify the smallest fragments of BDS-I, obtained by peptide synthesis, able to inhibit K_V_3.4 currents. For this purpose, whole-cell patch clamp technique was used to evaluate the effects of BDS-I fragments on K_V_3.4 currents in CHO cells heterologously expressing K_V_3.4. We found that BDS-I[1-8] fragment, containing the N-terminal octapeptide sequence of full length BDS-I, was able to inhibit K_V_3.4 currents in a concentration dependent manner, whereas the scrambled sequence of BDS-I[1-8] and all the other fragments obtained from BDS-I full length were ineffective. As we demonstrated in a previous study, BDS-I full length is able to counteract Aβ_1−42_-induced enhancement of K_V_3.4 activity, preventing Aβ_1−42_-induced caspase-3 activation and the abnormal nuclear morphology in NGF-differentiated PC-12 cells. Similarly to BDS-I, we found that BDS-I[1-8] blocking K_V_3.4 currents prevented Aβ_1−42_-induced caspase-3 activation and apoptotic processes. Collectively, these results suggest that BDS-I[1-8] could represent a lead compound to be developed as a new drug targeting K_V_3.4 channels.

## Introduction

Voltage-gated potassium (K_V_) channels are transmembrane proteins with strong selectivity for K^+^ ions and sensitivity to voltage changes. In neuronal cells, K_V_ channels are key regulators of membrane excitability, resting membrane potentials and spontaneous firing rate, action potential waveform and duration, neurotransmitter release, and apoptosis (Rudy et al., 1999). Several neuronal populations in the central nervous system (CNS) are required to generate action potentials with high frequency (Coetzee et al., 1999). K_V_ channels of the K_V_3 family are prominently expressed in these neurons and have a central role in facilitating sustained and/or repetitive high frequency firing (Moreno et al., 2001). The subunits of the K_V_3 *Shaw*-related subfamily (K_V_3.1-K_V_3.4), assembled into homotetramers with identical subunits or heterotetramers with different subunits, display unique biophysical properties such as high thresholds of activation, rapid activation and deactivation kinetics and relatively large conductance (Coetzee et al., 1999; Rudy et al., 1999). In rodents, three of four known K_V_3 genes (K_V_3.1-K_V_3.3) are conspicuously expressed in the CNS, whereas K_V_3.4 transcripts are abundant in skeletal muscle, sympathetic neurons, and weakly expressed in a few neuronal types in the brain in physiological conditions (Heinemann et al., 1996; Rudy et al., 1999). On the other hand, many evidence have been provided revealing the increase of K_V_3.4 protein expression in several pathological conditions such as hypoxia (Kaab et al., 2005), oxidative stress (Song et al., 2017), and neurodegeneration (Baranauskas et al., 2003; Angulo et al., 2004; Boscia et al., 2017).

Moreover, among the K_V_3 subfamily, only the K_V_3.4 channel carries the fast inactivating potassium currents I_A_ and exhibits peculiar pharmacological characteristics. In fact, similarly to the other K_V_3 channels, K_V_3.4-mediated currents are highly sensitive to external tetraethylammonium or 4-aminopyridine and, in addition, a selective responsiveness of K_V_3.4 to some toxins have been reported. In this regard, Diochot et al. reported that two 43-amino acid peptides from the sea anemone *Anemonia sulcata*, the blood depressing substance (BDS)-I and BDS-II, show a specific blocking activity against the I_A_ mediated by this channel (Diochot et al., 1998). These toxins directly inhibit K_V_3.4 channels in a reversible manner, with a similar IC_50_ (Diochot et al., 1998), despite a difference in the time-dependence of their effect has been suggested (Diochot et al., 1998; Song et al., 2017). Another aspect that deserves a mention is that the drug sensitivity of K_V_3.4 pore-forming α subunit, as well as its gating kinetics, conductance and ion selectivity, is modulated by the interaction with its accessory β subunit, the MinK-related peptide 2 (MiRP2) (Abbott et al., 2001; McCrossan and Abbott, 2004).

The I_A_ currents mediated by K_V_3.4 channel subunits have been recognized as a relevant player in Alzheimer's disease (AD) and are now emerging as a new target candidate for AD (Angulo et al., 2004; Pannaccione et al., 2005, 2007; Boda et al., 2012; Boscia et al., 2017). In previous studies, we demonstrated that β-amyloid peptide 1-42 (Aβ_1−42_) alters the properties of K^+^ currents in primary hippocampal neurons (Pannaccione et al., 2005) through the selective up-regulation of K_V_3.4 channels mediated by the activation of the transcriptional factor NF-κB (Pannaccione et al., 2007). Moreover, we also reported an increase in cell death caused by the reduction of cytoplasmic K^+^ concentrations, because of the enhanced expression and function of this K^+^ channel upon Aβ_1−42_ exposure (Pannaccione et al., 2007). In addition, it has been demonstrated the correlation between altered intracellular K^+^ concentrations and apoptotic processes (Yu, 2003). Interestingly, the concept that K_V_3.4 channels are involved in the Aβ_1−42_ neurotoxic effects (Abbott et al., 2001) was further supported by our results showing that BDS-I treatment, by blocking the K_V_3.4 channel, prevents the apoptotic cascade triggered by Aβ_1−42_ fragment in hippocampal neurons, thus exerting a potent neuroprotective action (Pannaccione et al., 2007). Importantly, these previous results identified the K_V_3.4 channel as a new molecular target, thus revealing a possible new strategy in the scenario of pharmacological therapies against AD progression.

For these reasons, the main goal of the present study has been to identify the smallest BDS-I amino acid sequence able to exert a neuroprotective effect by blocking K_V_3.4 activity and to provide a new opportunity in the development of potential drugs for AD treatment.

## Materials and Methods

### Materials

Fmoc-protected amino acids, Rink amide resin, and coupling reagents for SPPS compatibility, all were purchased from GL Biochem (Shanghai, China). Common solvents for SPPS and HPLC purification and characterization were obtained from VWR (Milano, Italy), whereas trifluoroacetic acid (TFA) was purchased from Iris Biotech GmbH (Marktredwitz, Germany). Other unmentioned materials were from Sigma Chemicals (St. Louis, MO, USA). Rabbit polyclonal anti-K_V_3.4, rabbit polyclonal anti-MiRP2, NGF 2.5S, TTX, nimodipine were from Alomone Labs (Jerusalem, Israel). Rabbit polyclonal anti-cleaved caspase-3 and rabbit monoclonal anti-Aβ were purchased from Cell Signaling (Massachusetts, USA). DMEM, FBS, non-essential amino acids, penicillin, streptomycin, and PBS were from Gibco-BRL (Grand Island, NY, USA). Lipofectamine 2000 was from Invitrogen Corp (Carlsbad, CA, USA). The Aβ_1−42_ peptide was synthesized by INBIOS (Pozzuoli, Naples, Italy). The BDS-I toxin and BDS-I fragments, BDS-I[1-8], BDS-I[1-8scr], BDS-I[7-14], BDS-I[13-20], BDS-I[19-26], BDS-I[25-32], BDS-I[31-38], BDS-I[37-44], were synthesized by Prof Paolo Grieco from Dept. of Pharmacy, University of Naples “Federico II,” Naples, Italy. Non-fat dry milk and precast gels 4-20% were from Bio-Rad Laboratories (Milan, Italy). Protease Inhibitor Cocktail II was from Roche Diagnostic (Monza, Italy). Glass coverslips were from Carolina Biological Supply Company (Burlington, NC, USA). The nitrocellulose membranes and ECL Western Detection Kit were from Amersham Bioscience (Buckinghamshire, UK).

### Heterologous Expression of K_V_3.4 and MiRP2 cDNAs

K_V_3.4 and MiRP2 channel subunits were expressed in Chinese hamster ovary (CHO) cells by transient transfection. CHO cells were grown in 100 mm plastic Petri dishes in Dulbecco's Modified Eagle Medium containing 10% fetal bovine serum (FBS), non-essential amino acids (0.1 mm), penicillin (50 U/ml), and streptomycin (50 μg/ml) in a humidified atmosphere at 37°C with 5% CO_2_ (Miceli et al., 2015). K_V_3.4 and MiRP2 cDNAs were kindly provided by the Professor Goldstein of the Department of Pediatrics and Institute for Molecular Pediatric Sciences, University of Chicago, USA. For electrophysiological experiments, cells were seeded on glass cover-slips (Glaswarenfabrik Karl Hecht KG, Sondheim, Germany) coated with poly-L-lysine (50 μg/mL) and transfected the next day with the appropriate cDNAs using Lipofectamine 2000 according to guidelines for transfection. We performed electrophysiological experiments 48 h after transfection.

### Cell Culture

Rat pheochromocytoma cells (PC-12 cells) were grown as previously described (Pannaccione et al., 2005, 2007). For all the experiments, cells were seeded at low density on glass cover-slips coated with poly-L-lysine (50 μg/ml). Differentiation of PC-12 cells was achieved by treatment with neuronal growth factor (NGF) 2.5 S (50 ng/ml) for 7–9 days (Greene and Tischler, 1976). For electrophysiological experiments, cells were seeded on glass cover-slips and used after 7–9 days of NGF.

### Whole-Cell Electrophysiology

Currents from CHO cells were recorded at room temperature 1-2 days after transfection. The extracellular solution contained the following (in mM): 138 NaCl, 5.4 KCl, 2 CaCl_2_, 1 MgCl_2_, 10 glucose and 10 HEPES, adjusted pH 7.4 with NaOH. The pipette solution contained (in mM) the following: 140 KCl, 2 MgCl_2_, 10 EGTA, 10 HEPES, 5 Mg-ATP, adjusted pH 7.4 with KOH (Miceli et al., 2015). K_V_3.4 currents were measured by applying voltage steps from −100 mV to +60 mV from a holding potential of −80 mV (Abbott et al., 2001). Moreover, the total outward K^+^ currents were recorded in NGF-differentiated PC-12 cells using the extracellular solution contained the following (in mM): 150 NaCl, 5.4 KCl, 3 CaCl_2_, 1 MgCl_2_, 10 HEPES, adjusted pH 7.4 with NaOH. The pipette solution contained the following (in mM): 140 KCl, 2 MgCl_2_, 10 HEPES, 10 glucose, 10 EGTA, and 1 Mg-ATP adjusted at pH 7.4 with KOH (Diochot et al., 1998). All experiments were performed in the presence of 50 nM tetrodotoxin (TTX) and 10 μ M nimodipine in the extracellular solution. To discriminate K^+^ current components appropriate electrophysiological protocols were used (Pannaccione et al., 2007). Steady-state activation and inactivation properties were measured by using appropriate protocols (Pannaccione et al., 2005). Capacitive currents were elicited by 5-mV depolarizing pulses from −80 mV and acquired at a sampling rate of 50 kHz. The capacitance of the membrane was calculated according to the following equation: C_m_ = τ_c_·I_o_/Δ*E*_m_ (1-I_∞_/I_o_), where C_m_ is membrane capacitance, τ_c_ is the time constant of the membrane capacitance, I_o_ is the maximum capacitance current value, Δ*E*_m_ is the amplitude of the voltage step, and I_∞_ is the amplitude of the steady-state current. K^+^ currents were recorded by patch-clamp technique in CHO and NGF-differentiated PC-12 cells using a commercially available amplifier (Axopatch 200B, Molecular Devices) and Digidata 1322A interface (Molecular Devices, USA). The whole-cell configuration of the patch-clamp technique was adopted using glass micropipettes with a resistance of 2.5-5.0 MΩ. Data were acquired and analyzed using the pClamp software (version 9, Molecular Devices).

### BDS-I Fragments Synthesis

Peptides BDS-I[1-8], BDS-I[13-20], BDS-I[19-26], BDS-I[25-32], BDS-I[31-38], BDS-I[37-43], and BDS-I[1-8scr] were designed following the mimotopic strategy (Table 1). All compounds were synthesized manually by adopting a standard solid-phase peptide synthesis (SPPS) using the Fmoc/tBu orthogonal strategy (Merrifield, 1963; Carpino and Han, 1970; Merlino et al., 2016). The use of the Wang resin 0.7 mmol/g (GL Biochem, Shanghai, China) as solid support in the entire synthesis resulted in amidated C-terminal peptides after TFA-mediated cleavage (TFA/TIS/H_2_O, 95:2.5:2.5, 3 h). The crude peptides were purified by reverse-phase HPLC (RP-HPLC, Shimadzu Nexera) on a preparative column (PhenomenexKinetex, C18 column, 150 × 21.2 mm, 5 μm, 100 Å) using a linear gradient of acetonitrile (0.1% TFA) in water (0.1% TFA), from 10 to 90% over 20 min, with a flow rate of 10.0 mL/min and UV detection at 220 nm. The purity (>98%) was ascertained by analytical RP-HPLC on a PhenomenexKinetex C18 column (150 × 4.6 mm, 5 μm, 100 Å) and molecular weights of all peptides were confirmed by LC-ESI MS system (Agilent Technology).

**Table 1:** Peptide library on BDS-I sequence. BDS-I[1-8], BDS-I[7-14], BDS-I[13-20], BDS-I[19-26], BDS-I[25-32], BDS-I[31-38], BDS-I[37-43], and BDS-I[1-8scr] peptides were designed following the mimotopic strategy.

**Code**	**Sequence**
BDS-I[1-43]	AAPCFCSGKPGRGDLWILRGTCPGGYGYTSNCYKWPNICCYPH
^*^BDS-I[1-43]	AAPAFASGKPGRGDLWILRGTAPGGYGYTSNAYKWPNIAAYPH
BDS-I[1-8]	H-Ala-Ala-Pro-Ala-Phe-Ala-Ser-Gly-OH
BDS-I[7-14]	H-Ser-Gly-Lys-Pro-Gly-Arg-Gly-Asp-OH
BDS-I[13-20]	H-Gly-Asp-Leu-Trp-Ile-Leu-Arg-Gly-OH
BDS-I[19-26]	H-Arg-Gly-Thr-Ala-Pro-Gly-Gly-Tyr-OH
BDS-I[25-32]	H-Gly-Tyr-Gly-Tyr-Thr-Ser-Asn-Ala-OH
BDS-I[31-38]	H-Asn-Ala-Tyr-Lys-Trp-Pro-Asn-Ile-OH
BDS-I[37-43]	H-Asn-Ile-Ala-Ala-Tyr-Pro-His-OH
BDS-I[1-8scr]	H-Gly-Ser-Ala-Phe-Ala-Pro-Ala-Ala-OH

The synthesis was based on BDS-I full length sequence replacing the cysteine in position 4, 6, 22, 32, 39, and 40 by alanine order to prevent the formation of disulphide bonds and to obtain unfolded peptides. We obtained a small library characterized by overlapping peptides of equal length, containing 8 amino acids, except for the sequence BDS-I[37-43].

* *BDS-I[1-43] with cysteine → lanine substitution*.

BDS-I[1-8]: AAPAFASG-OH, (3.2 mg, yield 31%, purity >98%), observed mass 690.75 (M+H)1+, calculated mass 691.32.

BDS-I[7-14]: GKPGRGDL-OH, (2.8 mg, yield 27%, purity >98%), observed mass 798.89 (M+H)1+, calculated mass 799.03.

BDS-I[13-20]: GDLWILRG-OH, (4.2 mg, yield 34%, purity >98%), observed mass 929.08 (M+H)1+, calculated mass 929.76.

BDS-I[19-26]: RGTAPGGY-OH, (3.4 mg, yield 25%, purity >98%), observed mass 777.83 (M+H)1+, calculated mass 778.21.

BDS-I[25-32]: GYGYTSNAY-OH, (5.7 mg, yield 38%, purity >98%), observed mass 995.00 (M+H)1+, calculated mass 995.67.

BDS-I[31-38]: NAYKWPNI-OH, (4.6 mg, yield 30%, purity >98%), observed mass 1005.13 (M+H)1+, calculated mass 1005.90.

BDS-I[37-43]: NIAAYPH-OH, (5.1 mg, yield 41%, purity >98%), observed mass 784.86 (M+H)1+, calculated mass 784.97.

BDS-I[1-8scr]: GSAFAPAA-OH, (2.4 mg, yield 43%, purity > 98%), observed mass 690.77 (M+H)1+, calculated mass 691.32.

### BDS-I Fragment Application

All BDS-I fragments, BDS-I[1-8], BDS-I[7-14], BDS-I[13-20], BDS-I[19-26], BDS-I[25-32], BDS-I[31-38], BDS-I[37-43] as well as the negative control peptide BDS-I[1-8scr] have been applied by acute treatment.

For the acute treatment, each peptide has been added in the extracellular solution. For each cell, we first recorded K^+^ currents as previously described, in the absence of peptides. After that, we switched from the extracellular solution without BDS-I peptides to the extracellular solution containing the BDS-I peptide. In these conditions, we recorded K^+^ currents until the steady-state was reached. This protocol has been performed to test the effect on K_V_3.4 currents of each BDS-I fragment as well as of BDS-I[1-8scr].

To evaluate the effect of the active fragments in the presence of Aβ_1−42_ oligomers, we pre-treated cells by adding the fragment in the culture medium at the final concentration of 100 nM, 30 min before exposing cells to Aβ_1−42_ oligomers and lasted for 24 h.

### Assessment of Nuclear Morphology

Nuclear morphology was evaluated by using the fluorescent DNA-binding dye Hoechst-33258 already described (Pannaccione et al., 2007).

### Western-Blot Analysis

NGF-differentiated PC-12 and CHO cells were washed in phosphate buffered saline (PBS) and collected by gentle scraping in ice-cold RIPA buffer containing in mM: 50 Tris pH 7.4, 100 NaCl, 1 EGTA, 1 PMSF, 1 sodium orthovanadate, 1 NaF, 0.5% NP-40, and 0.2% SDS supplemented with protease inhibitor cocktail II. After sonication and incubation for 1 h on ice, we centrifuged at 12,000 rpm at 4°C for 30 min and collected the supernatants. The protein content of resulting supernatant was determined using the Bradford reagent. Hundred microgram of proteins were mixed with a Laemmli sample buffer; then, they are applied and resolved on SDS-PAGE polyacrylamide gels. Following transfer onto nitrocellulose membranes, non-specific binding sites were blocked by incubation for 2 h at 4°C with 5% non-fat dry milk in TBS-T buffer; subsequently, incubated with primary antibodies overnight at 4°C. After three washes with TBS-T, the membranes were incubated 1 h with the appropriate secondary antibody. Excessive antibodies were then washed away three times (10 min) with TBS-T. Proteins were visualized with peroxidase-conjugated secondary antibodies, using the enhanced chemiluminescence system. The software Image J (NIH, Bethesda, MA, USA) was used for densitometric analysis. Primary antibodies were: rabbit polyclonal anti-K_V_3.4 (1:1,000), rabbit polyclonal anti-MiRP2 (1:1,000), rabbit polyclonal anti-cleaved caspase-3 (1:1,000) and mouse monoclonal anti-α-Tubulin (1:3,000).

### Aβ Treatments

The peptide used in our study was synthesized by INBIOS (Pozzuoli, Naples, Italy) using the Aβ_1−42_ sequence of human APP [UniProtKB-P05067 (A4_HUMAN)]. The purity of the peptide was assessed by high-performance liquid chromatography (>95% pure) and the amino acid composition was verified by mass spectrometry. The Aβ oligomers was resuspended in 1,1,1,3,3,3-hexafluoro-2-propanol (HFIP; Sigma, Milan, Italy), at a final concentration of 1 mM. Then, the preparation was aliquoted, microcentrifugeted, dried by SpeedVac and stored at −20°C. Before their use, Aβ oligomer aliquots were first resuspended in dimethyl sulfoxide (DMSO) at a final concentration of 5 mM, and then diluted in ice-cold cell culture medium (phenol red-free Ham's F-12, Sigma, Milan, Italy), to the final concentration of 100 μM. Next, after vortexing for 30 sec, the resuspension was incubated for 24 h at 4°C. Finally, the solution was centrifuged at 14,000 rpm at 4°C for 10 min, and the supernatant, containing Aβ_1−42_oligomers, aliquoted and stored at −20°C (Stine et al., 2003). Aβ_1−42_were added to the culture medium at the final concentration of 5 μM for 24 h. The pre-aggregated preparation of Aβ oligomers was analyzed in SDS-PAGE using the rabbit monoclonal anti-Aβ on precast gels 4-20%. Oligomers between 4 and 15 kDa were the major species of the Aβ oligomers in the preparation.

### Data and Statistical Analysis

GraphPad Prism 6.02 was used for statistical analyses (GraphPad Software, La Jolla, CA). The data are expressed as the mean ± S.E.M. of the values obtained from individual experiments. Statistical comparisons between groups were performed by Student's *t*-test or one-way analysis of variance (ANOVA) followed by Newman-Keuls' test; *p* < 0.05 was considered significant.

## Results

### Synthesis of BDS-I Fragments

To identify the key amino acidic residues of BDS-I essential for its inhibitory action on K_V_3.4 channels, we performed an overlapping peptide library on BDS-I sequence (Table 1). This small library is characterized by overlapping peptides of equal length, containing 8 amino acids, except for the sequence BDS-I[37-43]. Moreover, the cysteine in position 4, 6, 22, 32, 39, and 40 in the sequence of BDS-I[1-43], presumably involved in the tertiary structure of BDS-I, have been replaced by alanine in order to prevent the formation of disulphide bonds and to obtain unfolded peptides. This approach allowed us to focus on the importance of the amino acid sequence rather than the tertiary structure of BDS-I fragments.

### Screening of BDS-I Fragments ‘Activity on K_V_3.4 Currents by Patch Clamp Technique

**Figure 1:**
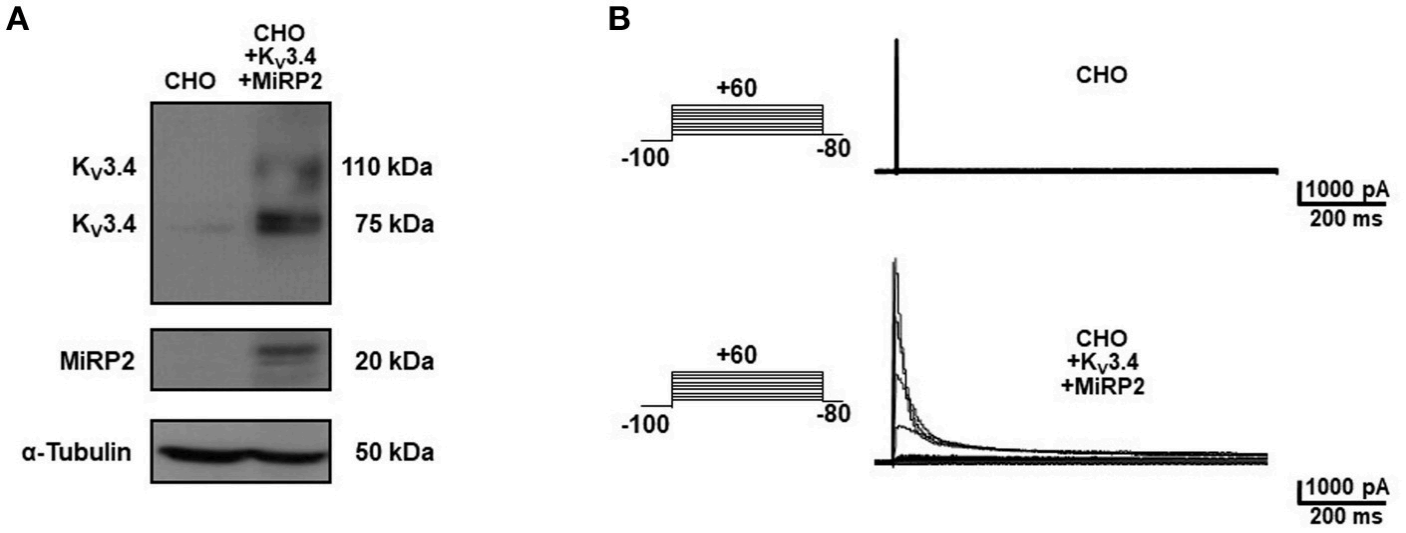
Heterologous expression of K_V_3.4 and MiRP2 cDNAs in CHO cells. **(A)** Representative western blot of K_V_3.4 and MiRP2 protein expression in CHO cells under control condition and transiently transfected with K_V_3.4 and MiRP2 cDNAs. **(B)** Representative traces of K_V_3.4 currents recorded in CHO cells under control condition and transiently transfected with K_V_3.4 and MiRP2 cDNAs.

**Figure 2:**
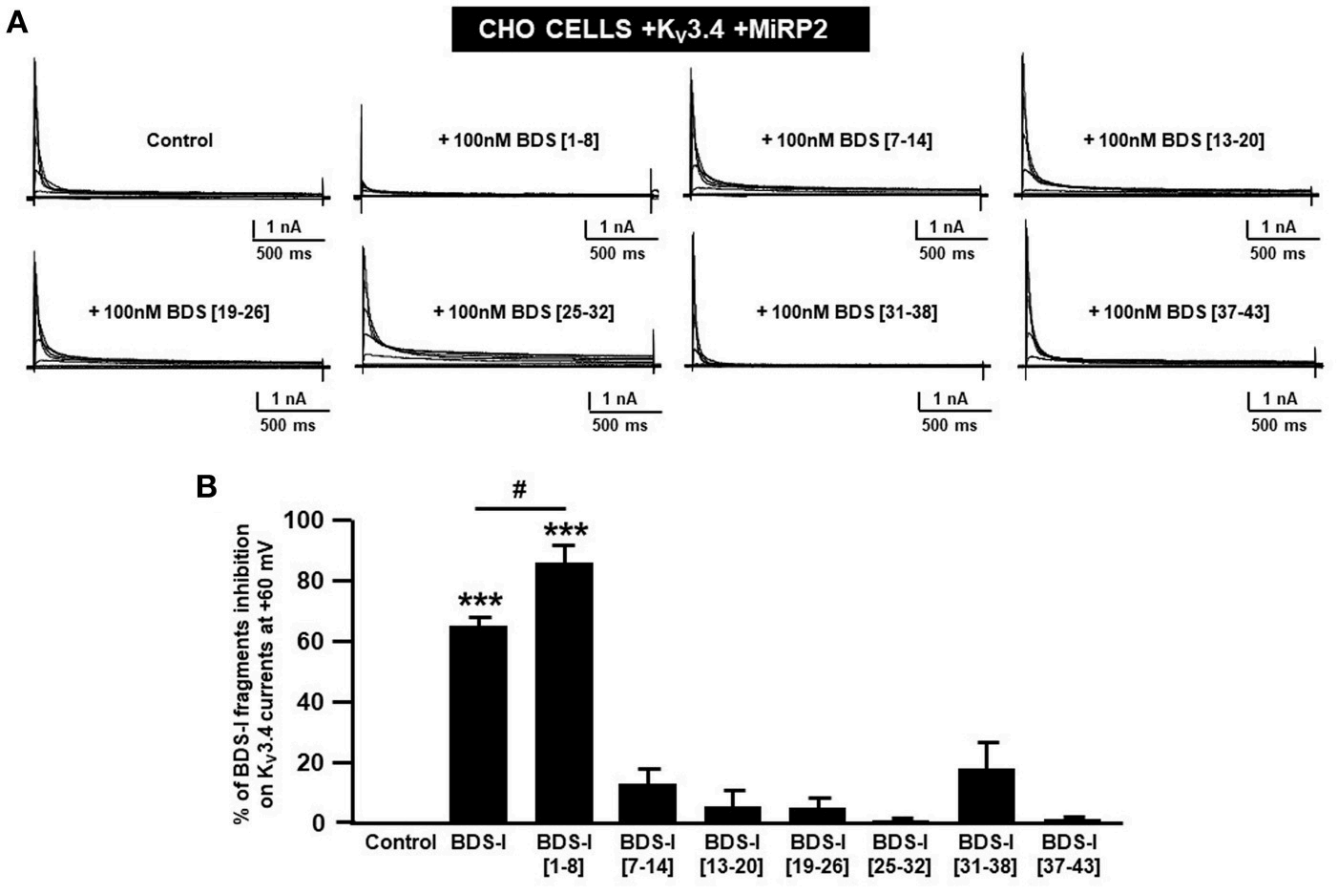
Screening of BDS-I fragments ‘activity on K_v_3.4 currents by patch clamp technique. **(A)** Representative traces of K_v_3.4 currents recorded in CHO cells transiently transfected with K_v_3.4\MiRP2 cDNAs 24 h after the transfection. Each cell has been recorded under control condition and after exposure to 100 nM of the indicated BDS-I fragments (BDS-I[1-8], BDS-I[7-14], BDS-I[13-20], BDS-I[19-26], BDS-I[25-32], BDS-I[31-38], BDS-I[37-43]) and full BDS-I, added to the extracellular solution. The switch to the extracellular solution containing each specific peptide represented the start of the treatment. For each groups the traces show K_v_3.4 currents elicited by depolarizing steps of increasing voltages from −80 to +60 mV (20 mV of increments), preceded by a conditioning prepulse at −100 mV. **(B)** Quantification of the % of inhibition of K_v_3.4 current densities exerted by BDS-I fragments, at the peak of the depolirizing pulse (+60 mV) represented in A. Quantification of K_v_3.4 currents inhibition. Values are expressed as % of inhibition, obtained as [(C_0_-C_1_)/C_0_] × 100 where C_0_ is K_v_3.4 control current; C_1_ is K_v_3.4 current in the presence of BDS-I fragments. Values are expressed as percentage mean±SEM from 6 to 10 cells *per* experimental group of three independent experimental sessions. Statistical comparisons between groups were performed by one-way ANOVA followed by Newman-Keuls' test; ***p < 0.001 vs. control; #*p* < 0.01 vs. BDS-I full length.

**Figure 3:**
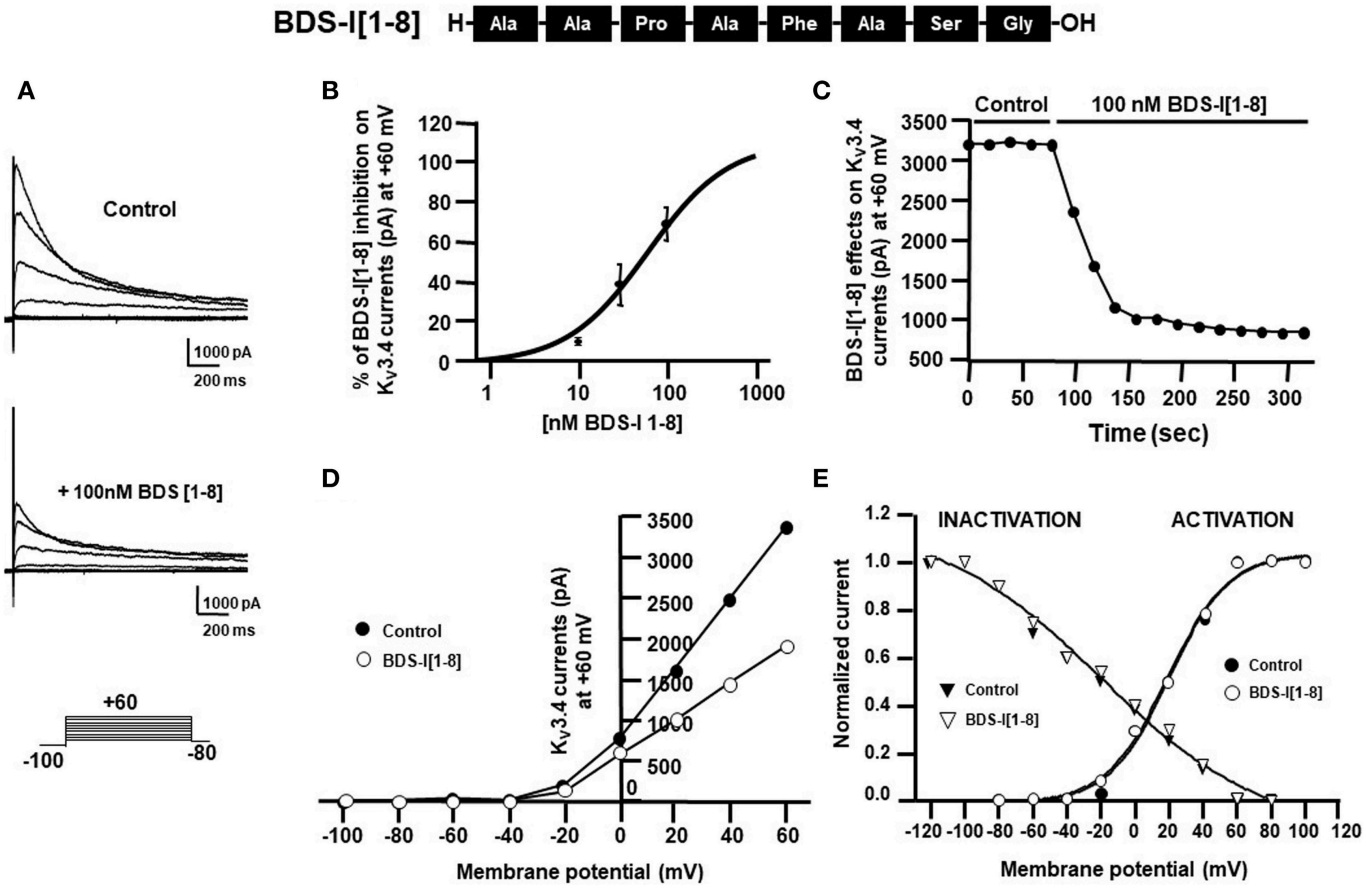
Effect of BDS-I[1-8] fragment on K_V_3.4 currents in CHO cells transiently transfected with KV3.4 and MiRP2 **(A)** Representative traces of K_V_3.4 currents recorded in CHO cells under control condition and in the presence of BDS-I[1-8] (100 nM). **(B)** Inhibitory dose-response curves to determine the IC_50_ for BDS-I[1-8] on K_V_3.4 currents. The inhibitory dose-response curve data were plotted as % of inhibition normalized to controls with applied curve fits calculated using GraphPad Prism. **(C)** Time-course of outward K^+^ currents recorded in CHO cells transfected with K_V_3.4 and MiRP2 exposed to 100 nM BDS-I[1-8]. The currents were measured at the peak of repetitive (20 sec) depolarizing pulses (+60 mV) were plotted vs. time. **(D)** Representative current-voltage (I-V) relationship for K_V_3.4 peak current measured before and during application of 100 nM BDS-I[1-8]. Currents were induced by a set of depolarizing pulses from +60 to −100 mV (in 20 mV steps). **(E)** Representative steady-state activation and inactivation curves of K_V_3.4 channels under control condition and in the presence of BDS-I[1-8] (100 nM). For the voltage-dependent activation the currents recorded upon repolarization to −100 mV were measured, normalized to the maximum value, and plotted vs. the membrane voltage of the depolarizing step. For the inactivation curves, the initial currents recorded immediately after delivering the +80-mV test pulse were measured, normalized to the maximum value, and plotted vs. the membrane voltage of the conditioning pulses. The experimental data were fitted to the following form of the Boltzmann equation: *gK*_v_ = max*/*(1 + exp(*V*12-*V*)/*k*), where *V* is the test potential, *V*12 is the half-activation potential, and *k* (or *kT*/*ze*) is the slope of the conductance to voltage relationship.

To identify the smallest BDS-I amino acid sequence able to block K_v_3.4 activity, we evaluated the effect of the newly synthesized BDS-I fragments in CHO cells transiently transfected with K_v_3.4/MiRP2, by patch clamp technique in whole cell configuration. K_v_3.4 expression and activity in transfected CHO cells were monitored by western blot analysis and electrophysiological experiments, respectively (Figures 1A,B). After acute application of the peptides, we found that only BDS-I[1-8] fragment (100 nM), containing the N-terminal octapeptide sequence of full length BDS-I, similarly to the full length, was able to inhibit K_v_3.4 currents (Figures 2A,B), whereas the other BDS-I fragments, BDS-I[7-14], BDS-I[13-20], BDS-I[19-26], BDS-I[25-32], BDS-I[31-38], and BDS-I[37-44] (100 nM) did not (Figures 2A,B, 3A). The percentage of inhibition of K_v_3.4 currents exerted by BDS-I[1-8] fragment (100 nM) was more than 80%. Interestingly, BDS-I, displaying a percentage of inhibition of **~**60%, appeared less effective than BDS-I[1-8] fragment (Figure 2B). Moreover, we found that BDS-I[1-8] reduced K_v_3.4 currents in a concentration-dependent manner, with an IC_50_ value of 75 nM (Figure 3B). The time-course of the effect of BDS-I[1-8] on K_v_3.4 currents showed that the steady-state of inhibition was reached at 150 s of peptide exposure (Figure 3C). Inhibition of the K_v_3.4 current induced by BDS-I[1-8] was not voltage-dependent as evidenced by I-V relationship for K_v_3.4 peak current measured before and during application of 100 nM BDS-I[1-8] (Figure 3D). Furthermore, BDS-I[1-8] treatment did not modify the steady-state parameters for the activation and inactivation of K_v_3.4 currents (Figure 3E).

### Effect of BDS-I[1-8] Scrambled Peptide on K_**v**_3.4 Currents

**Figure 4:**
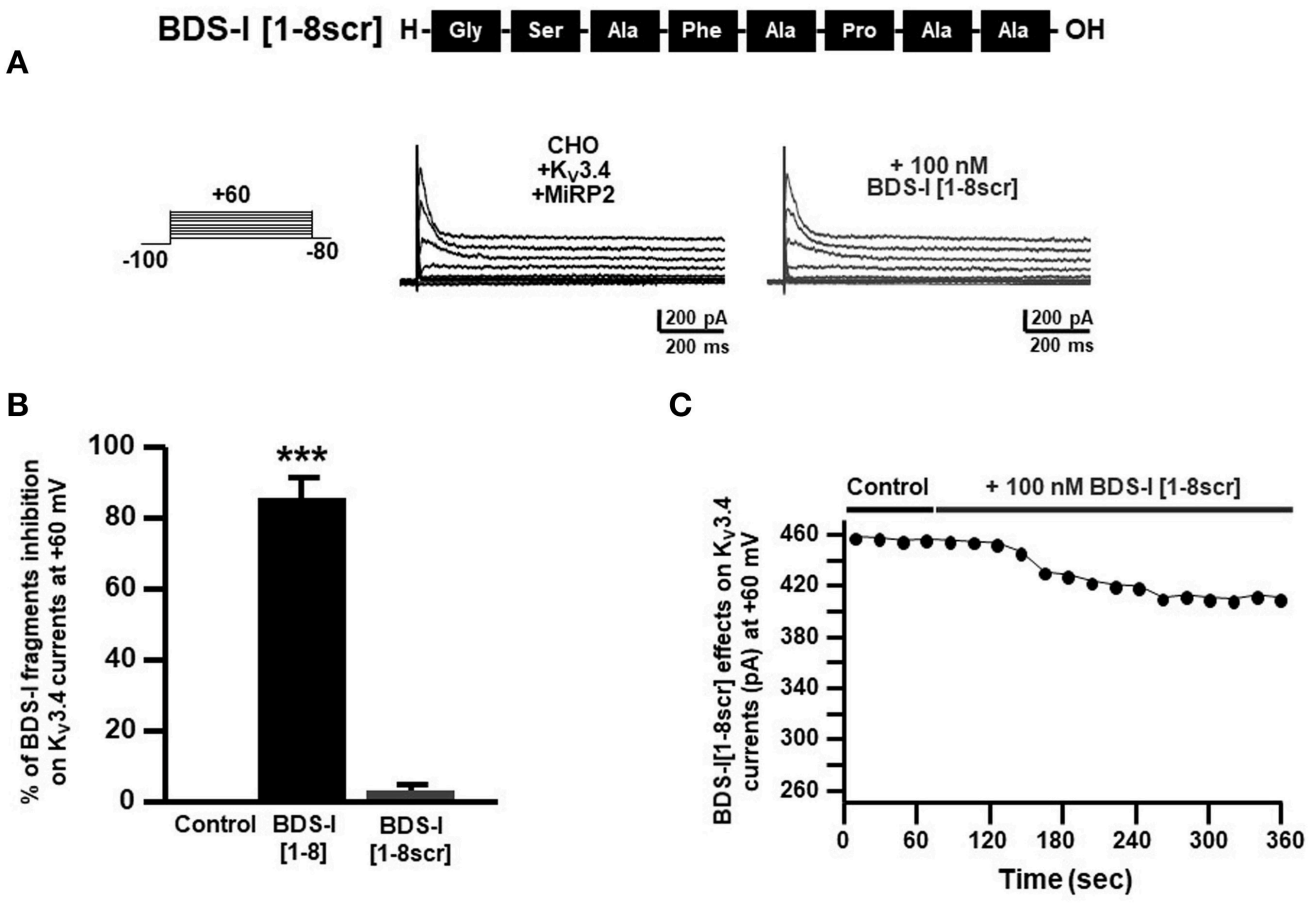
Effect of BDS-I[1-8scr] fragment on K_v_3.4 currents in CHO cells transfected with K_v_3.4 and MiRP2. **(A)** Representative traces of K_v_3.4 currents recorded in CHO cells under control condition and in the presence of BDS-I[1-8scr] 100 nM. Each cell has been recorded under control condition and after exposure to 100 nM of BDS-I[1-8scr] added to the extracellular solution. The switch to the extracellular solution containing each specific peptide represented the start of the treatment. For each groups the traces show K_v_3.4 currents elicited by depolarizing steps of increasing voltages from −80 to +60 mV (20 mV of increments), preceded by a conditioning prepulse at −100 mV. **(B)** Quantification of the % of inhibition of K_v_3.4 current densities exerted by BDS-I fragments, at +60 mV represented in A. Quantification of K_v_3.4 current inhibition. Values are expressed as % of inhibition, obtained as [(C_0_-C_1_)/C_0_] × 100 where C_0_ is K_v_3.4 control current; C_1_ is K_v_3.4 current in the presence of BDS-I[1-8scr]. Values are expressed as percentage mean±SEM from 6 to 10 cells *per* experimental group of 3 independent experimental sessions. Statistical comparisons between groups were performed by one-way ANOVA followed by Newman-Keuls' test; ***p < 0.001 vs. control. **(C)** Time-course of outward K^+^ currents recorded in CHO cells transfected with K_v_3.4 and MiRP2 exposed to 100 nM BDS-I[1-8scr]. The currents were measured at the peak of repetitive (20 s) depolarizing pulses (+60 mV) were plotted vs. time.

To confirm that the inhibitory effect of BDS-I[1-8] on K_V_3.4 currents was sequence-specific, we synthesized a peptide with the scrambled sequence of BDS-I[1-8], named BDS-I[1-8scr]. Electrophysiological experiments performed in CHO cells transiently transfected with K_V_3.4/MiRP2 showed that BDS-I[1-8scr] at the same concentration of 100 nM was not able to modify K_V_3.4 activity (Figures 4A,B). The time-course of current inhibition during BDS-I[1-8scr] exposure is presented in Figure 4C.

### Effect of BDS-I[1-8] on Aβ_1−42_-Induced Upregulation of K_**v**_3.4 Activity and Caspase-3 Activation in NGF-Differentiated PC-12 Cells

**Figure 5:**
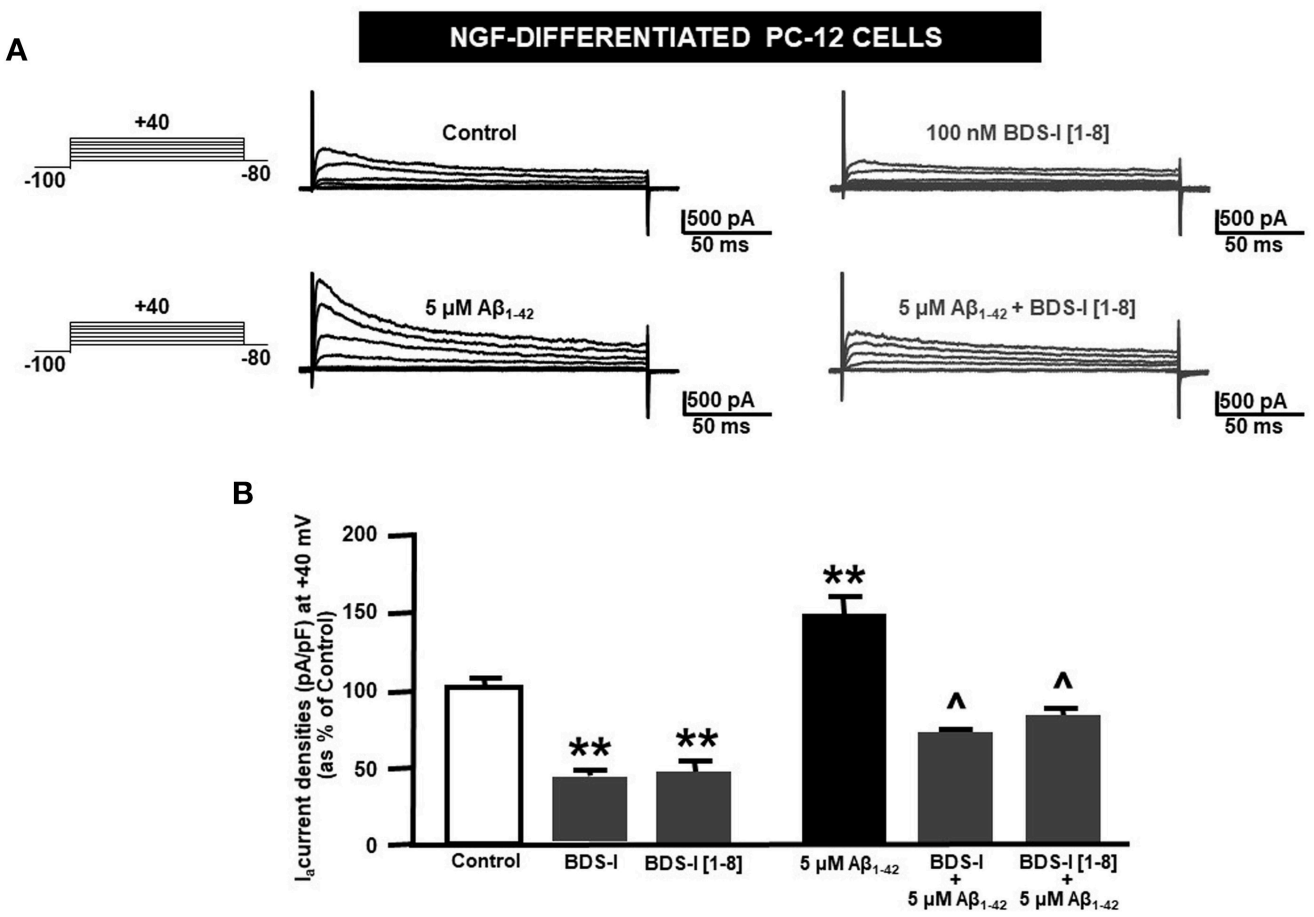
Effect of BDS-I[1-8] fragment on K_V_3.4 currents in NGF-differentiated PC-12 cells under control condition and exposed to Aβ_1−42_. **(A)** Representative traces of K_V_3.4 currents recorded in NGF-differentiated PC-12 cells under control condition and exposed to 100 nM of BDS-I[1-8] (top); representative traces of K_V_3.4 currents recorded in NGF-differentiated PC-12 cells exposed to Aβ_1−42_ oligomers (5 μM for 24 h) in the absence and in the presence of BDS-I[1-8] fragment (30 min of pre-treatment before adding Aβ_1−42_ oligomers) (bottom). For each group, traces show K_V_3.4 currents elicited by depolarizing steps of increasing voltages from −80 to +40 mV (20 mV of increments), preceded by a conditioning prepulse at −100 mV. **(B)** Quantification of K_V_3.4 current densities, at the peak of the depolirizing pulse (+40 mV) represented in A. Values are expressed as percentage mean±SEM from 6 to 10 cells *per* experimental group of 3 independent experimental sessions. Statistical comparisons between groups were performed by one-way ANOVA followed by Newman-Keuls' test; **p < 0.01 vs. control; ∧ *p* < 0.01 vs. Aβ_1−42_.

**Figure 6:**
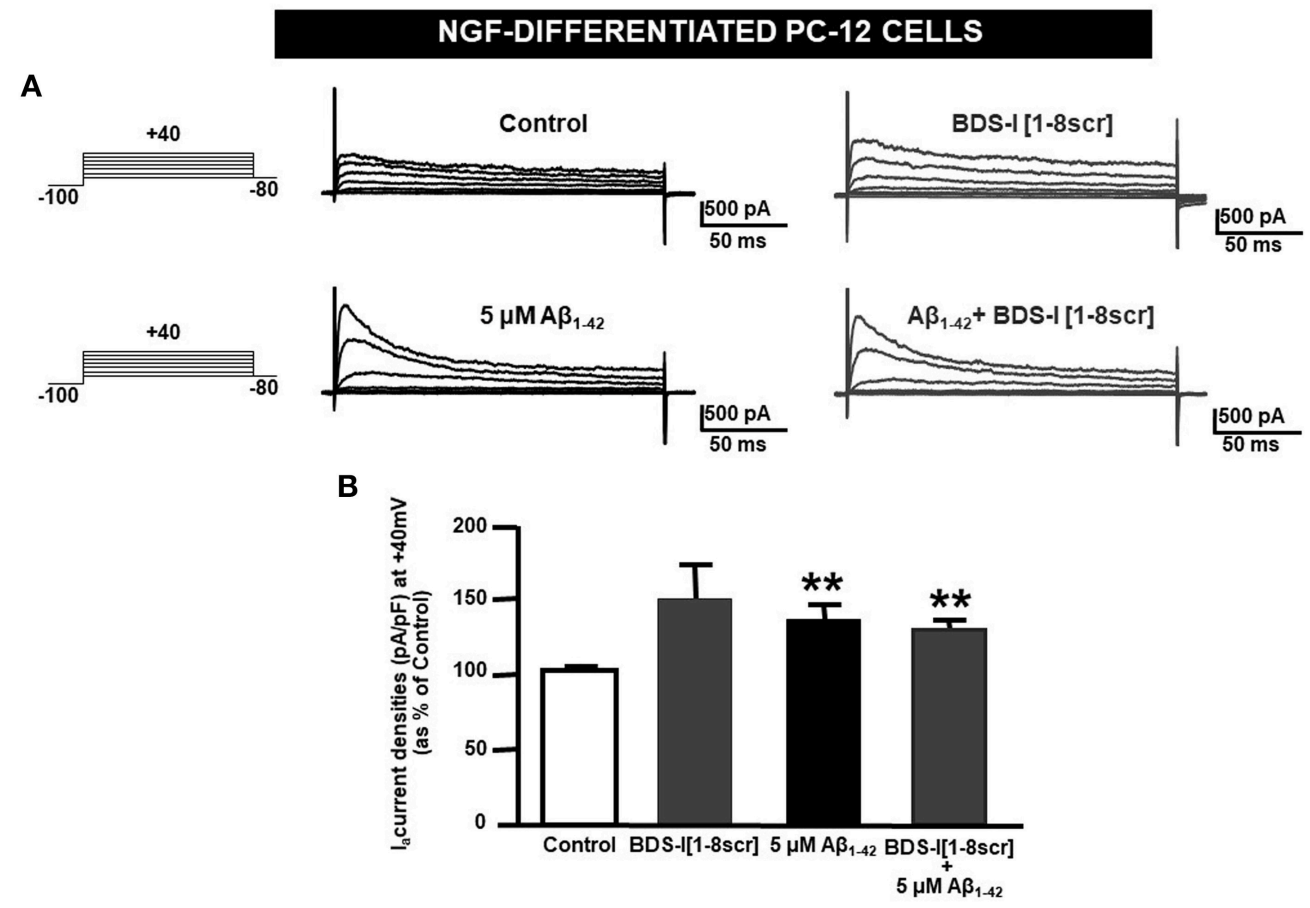
BDS-I[1-8scr] fragment on K_V_3.4 currents in NGF-differentiated PC-12 cells. **(A)** Representative traces of K_V_3.4 currents recorded in NGF-differentiated PC-12 cells under control condition and exposed for 24 h to 100 nM BDS-I[1-8scr] (top); representative traces of K_V_3.4 currents recorded in NGF-differentiated PC-12 cells exposed to Aβ_1−42_ oligomers (5 μM, 24 h) in the absence and in the presence of BDS-I[1-8scr] fragment (30 min of pre-treatment before adding Aβ_1−42_ oligomers) (bottom). For each groups the traces show K_V_3.4 currents elicited by depolarizing steps of increasing voltages from −80 to +40 mV (20 mV of increments), preceded by a conditioning prepulse at −100 mV. **(B)** Quantification of K_V_3.4 current densities, at the peak of the depolirizing pulse (+40 mV) represented in A. Values are expressed as percentage mean±SEM from 6 to 10 cells per experimental group of 3 independent experimental sessions. Statistical comparisons between groups were performed by one-way ANOVA followed by Newman-Keuls' test; **p < 0.01 vs. control.

**Figure 7:**
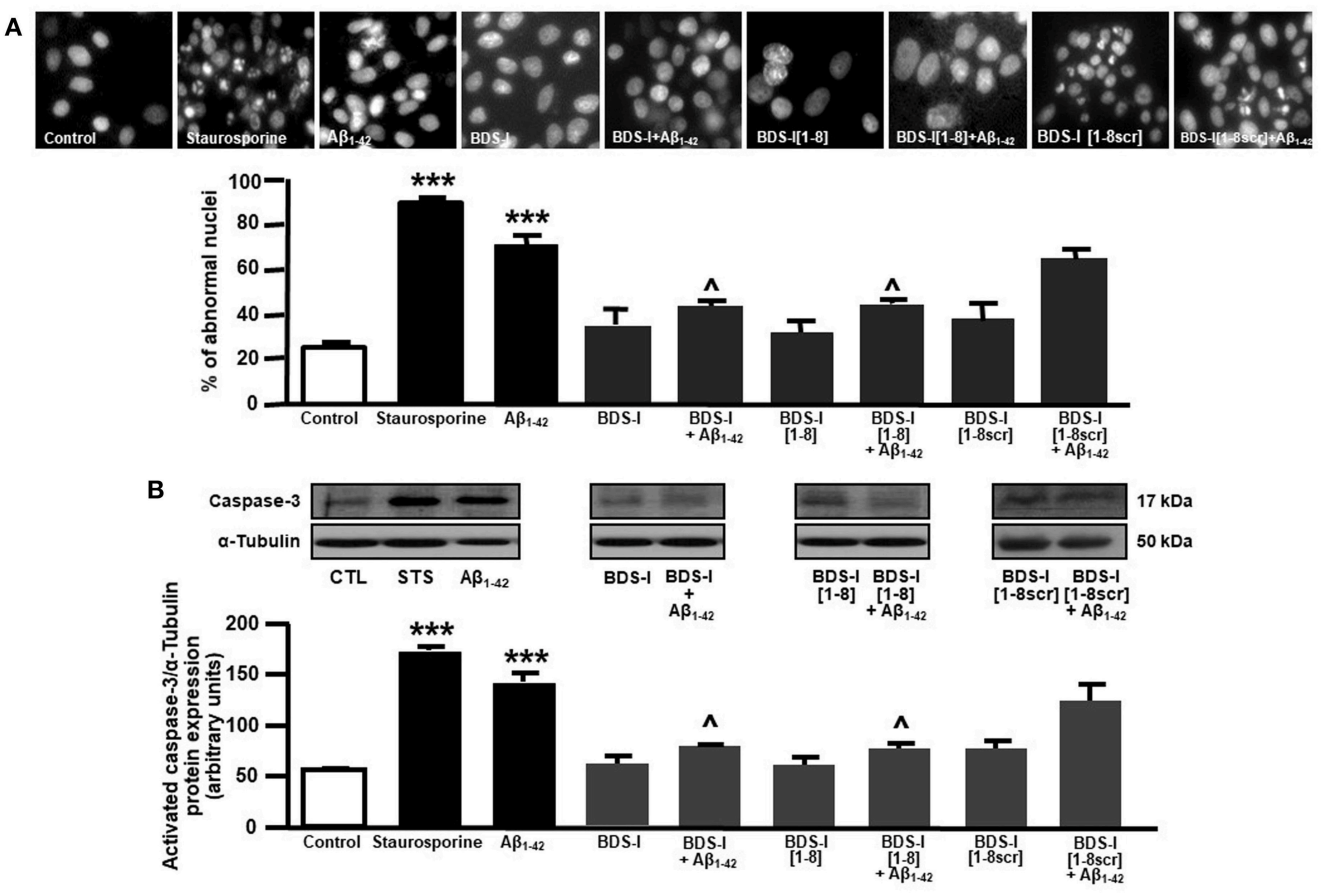
Effect of BDS-I[1-8] on Aβ_1−42_-induced abnormal nuclear morphology and caspase-3 activation in NGF-differentiated PC-12 cells. **(A)** Assessment of nuclear morphology with Hoechst-33258 in NGF-differentiated PC-12 cells under control condition and exposed to Aβ_1−42_ oligomers (5 μM, 24 h) in the absence and in the presence of BDS-I[1-8] fragment, BDS-I[1-8scr] fragment, BDS-I full length (3 h of pre-treatment before adding Aβ_1−42_ oligomers) (top). Quantification of the percentage of abnormal nuclei in NGF-differentiated PC-12 cells under control condition and exposed to Aβ_1−42_ oligomers (5 μM, 24 h) in the absence and in the presence of BDS-I[1-8] fragment, BDS-I[1-8scr] fragment, BDS-I full length (30 min of pre-treatment before adding Aβ_1−42_ oligomers) obtained in 3 separate experiments in which at least 10 microscopic fields were analyzed (~1,000 cells per group) (Bottom). Statistical comparisons between groups were performed by one-way ANOVA followed by Newman-Keuls' test; ***p < 0.001 vs. control; ∧ *p* < 0.01 vs. Aβ_1−42_. **(B)** Representative western blot (top) and densitometric quantification (bottom) of caspase-3 protein expression in NGF-differentiated PC-12 cells under control condition and exposed to Aβ_1−42_ oligomers (5 μM, 24 h) in the absence and in the presence of BDS-I[1-8] fragment, BDS-I[1-8scr] fragment, BDS-I full length (30 min of pre-treatment before adding Aβ_1−42_ oligomers). Values are expressed as mean±SEM of 3 independent experimental sessions. Statistical comparisons between groups were performed by one-way ANOVA followed by Newman-Keuls' test; ***p < 0.001 vs. control; ∧ *p* < 0.01 vs. Aβ_1−42_.

Analogously to the results obtained in CHO cells, BDS-I[1-8] fragment blocked K_V_3.4 activity in NGF-differentiated PC-12 cells (Figures 5A,B). Importantly, like BDS-I, BDS-I[1-8] fragment prevented the K_V_3.4 upregulation elicited by Aβ_1−42_ exposure (5 μM for 24 h) (Figures 5A,B). By contrast, BDS-I[1-8scr] did not counteract Aβ_1−42_-induced K_V_3.4 upregulation (Figures 6A,B). More interestingly, nuclear morphological studies by bisbenzimide Hoechst staining highlighted that, similar to the full length BDS-I, BDS-I[1-8], but not BDS-I[1-8scr], was able to significantly prevent the abnormal nuclear morphology induced by Aβ_1−42_ exposure in NGF-differentiated PC-12 cells (Figures 7A,B). These results were confirmed by western blot analysis of activated caspase-3. In fact, BDS-I[1-8] prevented Aβ_1−42_-induced caspase-3 activation whereas BDS-I[1-8scr] did not (Figure 7C).

## Discussion

In this study, a novel peptide obtained from the sequence of the sea anemone toxin BDS-I has been successfully identified as a new inhibitor of the K_V_3.4 channel subunits. In fact, BDS-I[1-8] fragment, containing the N-terminal octapeptide sequence of full length BDS-I, was able to inhibit K_V_3.4 currents in a concentration dependent manner, with an IC_50_ value of 75 nM, in CHO cells transiently transfected with K_V_3.4/MiRP2. Interestingly, the other BDS-I fragments, BDS-I[7-14], BDS-I[13-20], BDS-I[19-26], BDS-I[25-32], BDS-I[31-38], BDS-I[37-44], failed to modulate K_V_3.4 activity, thus confirming the key role played by the N-terminal octapeptide sequence of BDS-I in its pharmacological effect on K_V_3.4 currents. To confirm and validate the results obtained with BDS-I[1-8], we synthesized a scramble sequence of this peptide, as reported in Table 1. Importantly, the negative control peptide, BDS-I[1-8scr], did not display any pharmacological activity, thus highlighting that the sequence of BDS-I[1-8] is essential for the inhibitory effect of BDS-I on K_V_3.4 activity. The BDS-I[1-8], similarly to BDS-I, was able to selectively inhibit the fast inactivating K^+^ currents carried by the K_V_3.4 also in NGF-differentiated PC-12 cells, a well-known *in vitro* model mimicking the neuronal system, whereas BDS-I[1-8scr] did not exert any effect. More interestingly, BDS-I[1-8], but not BDS-I[1-8scr], fully counteracted the Aβ_1−42_-induced enhancement of K_V_3.4 activity and prevented Aβ_1−42_-induced caspase-3 activation and abnormal nuclear morphology. Indeed, we previously demonstrated that Aβ_1−42_ peptide induces a significant increase in K_V_3.4 expression and activity in NGF-differentiated PC-12 cells and hippocampal neurons and that BDS-I, by blocking K_V_3.4 channels, prevents the apoptotic cascade triggered by Aβ_1−42_ (Pannaccione et al., 2005, 2007). The present study clearly shows that, similarly to BDS-I, BDS-I[1-8] is able to exert a neuroprotective effect by counteracting the upregulation of K_V_3.4 channel induced by Aβ_1−42_.

Importantly, it has been widely demonstrated that the dysregulation of K_V_3.4 channel subunits contributes to neuronal and glial alterations in AD. Previous evidence has shown that the overexpression of K_V_3.4 channel subunits, occurring both in the early and in the advanced stages of the disease, is crucially involved in the development of the disease (Angulo et al., 2004). K_V_3.4 upregulation has also been related to amyloid pathology, as high levels of these subunits have been detected in amyloid plaques in post-mortem human brain tissues (Angulo et al., 2004). Accordingly, we recently demonstrated that the expression and function of K_V_3.4 channel subunits are precociously upregulated in cultured astrocytes exposed to Aβ oligomers and in reactive astrocytes of AD Tg2576 mice (Boscia et al., 2017). Indeed, our results suggest that the upregulation of K_V_3.4 channel subunits in astrocytes may be an early event in the AD brain, since we observed astrocytes accumulating Aβ and overexpressing K_V_3.4 subunits in cortex, hippocampus, and cerebellum of 6 month old Tg2576 mice (Boscia et al., 2017). All together, these evidence identify K_V_3.4 channels as a crucial player and, hence, as a possible pharmacological target in AD. On the other hand, K_V_3.4 has been implicated in several diseases, including cancer and cardiovascular pathologies. In particular, it has been reported that K_V_3.4 channels play a key role in cancer cell migration and invasion as BDS, similarly to K_V_3.4 silencing, was able to reduce the number of invasive cells (Song et al., 2018). Moreover, K_V_3.4 currents have been implicated in the proliferation of vascular smooth muscle cells, a crucial factor underlying the unwanted remodeling of arterial walls in pathological conditions (Miguel-Velado et al., 2010). In this scenario, the identification of the key residues of BDS-I essential for the inhibition of K_V_3.4 currents, namely BDS-I[1-8] peptide, provides a possible therapeutic opportunity for several diseases, despite subsequent approaches are needed to test BDS-I[1-8] stability and efficacy *in vivo*. Indeed, this small peptide could be a suitable lead compound for the development of new therapies targeting K_V_3.4 not only in neurodegeneration and neuroinflammation, but also in cancer and other pathological conditions.

## Data Availability

The raw data supporting the conclusions of this manuscript will be made available by the authors, without undue reservation, to any qualified researcher.

## Author Contributions

AP conceived the study. AP and RC designed the experiments and performed data analysis. PG and FM synthetized BDS-I fragments. RC and IP performed all the experiments. RC and IP wrote the manuscript. LA, AP, and PG reviewed and edited the final draft of the manuscript.

### Conflict of Interest Statement

The authors declare that the research was conducted in the absence of any commercial or financial relationships that could be construed as a potential conflict of interest. The handling editor declared a shared affiliation, though no other collaboration, with the authors RC, IP, PG, FM, LA, AP at time of review.
